# Review of Pulsation Signal Detection and Applications in Dynamic Photoacoustic Imaging

**DOI:** 10.3390/bios15090591

**Published:** 2025-09-08

**Authors:** Wenhan Zheng, Chuqin Huang, Jun Xia

**Affiliations:** Department of Biomedical Engineering, University at Buffalo, The State University of New York, Buffalo, NY 14260, USA; wzheng26@buffalo.edu (W.Z.); chuqinhu@buffalo.edu (C.H.)

**Keywords:** photoacoustic imaging, hemodynamics, pulsatile signal monitoring, PPG

## Abstract

Pulsatile signal detection plays an important role in monitoring various physiological parameters, primarily heart rate and blood oxygen saturation. Their applications range from clinical settings to personal health and wellness monitoring. PPG (photoplethysmography) can provide non-invasive optical measurements to detect blood volume changes in peripheral tissues. Yet, it suffers from low spatial resolution to precisely detect the pulsatile signal originating over 2 mm in human tissue. Ultrasound (US) provides a deep detectable range compared to the pure optical method. However, its low contrast to red blood cells and cluster artifacts makes it only detect the indirect pulsation from the surrounding tissue of blood vessels. Recent advances in PA imaging show its capability to precisely measure pulsatile signals originating from blood vessels in deep regions (over 10 mm) and its potential to accurately record blood oxygen saturation with high spatial and temporal resolution. This review article summarizes studies on photoacoustic (PA) pulsatile signal monitoring, highlights the technical advances, and compares it against optical and ultrasonic approaches.

## 1. Introduction

### 1.1. Background of Pulsatile Signal Detection

In vivo pulsatile signal detection is a scientific and medical technique used to monitor and study various physiological processes that exhibit rhythmic or pulsatile behavior within living organisms, typically humans or animals [1]. This technique involves the measurement and analysis of signals or changes in physiological parameters over time, focusing on capturing the periodic variations occurring naturally within the body [2,3]. The significance of in vivo pulsatile signal detection lies in its ability to provide valuable insights into several important aspects of biology and medicine, including cardiovascular research, hemodynamics, and respiratory studies.

The detected pulsatile signal can help researchers monitor heart rate and arterial pulse waveforms, providing critical information for cardiovascular health assessment, disease diagnosis, and the evaluation of treatment [4,5]. By acquiring pulsatile signals in blood vessels, researchers can study blood flow dynamics, identify abnormalities such as atherosclerosis or vascular stenosis, and assess the impact of interventions like medications or surgeries on blood circulation [6,7,8]. In addition, pulsatile signal detection can be employed to study respiratory patterns and lung function. It allows researchers to assess various parameters, such as the breathing rate and the regularity of respiration cycles [9]. Moreover, pulsatile detection plays a critical role in tissue perfusion diagnosis, as it provides dynamic information such as pulse amplitude, waveform morphology, and temporal variations across the tissue [10]. These parameters reflect the integrity of the arterial supply and can indicate occlusion or impaired microcirculation [11]. By mapping out the pulsatile variation in tissue and assessing localized hemodynamic responses, clinicians will have a more accurate evaluation of tissue perfusion status, which is essential for diagnosing ischemic conditions, monitoring wound healing, and guiding interventions in perfusion-related diseases [12]. In general, in vivo pulsatile signal allows researchers and clinicians to gain insights into the dynamic nature of physiological processes, leading to improved diagnosis, treatment, and understanding of various diseases and biological systems.

### 1.2. Existing Detection Approaches of Pulsatile Signals

Using optical approach to extract pulsatile signal from human tissue has been widely investigated for decades [13]. PPG is an optical technique which monitors optical absorption difference over time, resulting from variations in blood volume within the imaging region [14]. Typically, the PPG device comprises a light source and a photodiode. The light illuminates into the human tissue and the photodiode captures the fluctuation in light amplitude over time, revealing the pulsatile signal from the subject. Kao et al. [15] designed and validated a new PPG module for high-accuracy biomedical sensing. The module contains four light emitting diodes (LEDs) in different wavelengths. The PPG signal was optimized by achieving a simulated optical model under user’s skin, maximizing the ratios of pulsatile to non-pulsatile components in PPG waveforms. The optimized PPG device enables high accuracy in blood pressure estimation. However, the proposed method assumed PPG signal captured from longer wavelengths only contains deep region signal, which is not true in this case. Lai et al. [16] utilized imaging photoplethysmography (iPPG) to detect the perfusion levels of the microvasculature tissue bed in different perfusion conditions during intestinal surgeries. iPPG perfusion maps were successfully extracted from the intestine microvasculature, demonstrating that iPPG can be successfully used for detecting perturbations and perfusion changes in intestinal tissues during surgery. While iPPG possesses real-time imaging with a large field of view, the detectable depth of image acquisition is limited to superficial tissue, resulting in a restricted capture of perfusion information from deeper tissue layers. Another optical-based method called diffuse speckle pulsatile flowmetry (DSPF) is also applied to capture tissue-level pulsatile blood flow. For instance, Bi et al. [17] developed a portable DSPF to assess tissue ischemia status in patients with peripheral artery disease (PAD). While DSPF achieves slightly better imaging depth than PPG, it measures speckle decorrelation related to red blood cell motion and does not provide direct contrast to hemodynamics [18]. In addition, rhythmic contractions of blood vessels containing smooth muscle are often independent of cardiac pulsation or respiratory rhythm. When using PPG-based techniques to capture pulsatile signals, vasomotion within the imaging region may introduce perturbations [19].

Apart from optical methods, there have been explorations into utilizing ultrasonic approaches for extracting pulsatile signals. Jimenez et al. [20] presented a method using pulsatile signals from temporal ultrasonic imaging for continuous, noninvasive blood pressure monitoring. The method uses ultrasound imaging to measure arterial dimension variation over time, which provides pulsatile signal containing cross-sectional area information. In the meanwhile, the artery wall resonances are measured to further assist the blood pressure estimation without calibration. Wang et al. [21] proposed an ultrasonic device that can continuously monitor the central blood pressure (CBP) waveform from deeply embedded vessels. The device is ultrathin, stretchable, and conformal to the skin, enabling non-invasive, continuous, and accurate monitoring of cardiovascular events from multiple body locations. While these ultrasonic approaches offer a deeper imaging depth than optical methods, they measure the pulsatile waveform indirectly from the vessel wall movement [22]. Therefore, the method is mainly applied to large arteries, which do not need high spatial resolution.

### 1.3. Introduction to Photoacoustic Imaging

Photoacoustic imaging (PAI) takes advantage of both optics and ultrasound. The optical contrast is detected by a nanosecond laser pulse and transmitted acoustically. Therefore, it provides high spatial resolution without compromising the detectable depth due to the relatively weak acoustic scattering in human tissue [23,24]. In the meanwhile, the temporal resolution of PAI mainly depends on the pulse repetition frequency (PRF) of the laser and the arrival time of acoustic waves from the subject to the receiver [25]. Hence, precise pulsatile signals from deep regions with high spatial resolution can be acquired by applying a high PRF laser. To date, PAI has two major implementations [26]. The first, known as photoacoustic microscopy (PAM), provides high-resolution imaging and image reconstruction is typically not needed [27]. Based on the determining factor of spatial resolution, PAM can be further categorized into two subtypes: optical-resolution PAM (OR-PAM) and acoustic-resolution PAM (AR-PAM). The second PAI implementation, commonly known as photoacoustic computed tomography (PACT), utilizes wide-field light illumination and acoustic detection through a multi-element ultrasound transducer array. Due to the wide acceptance angle of the transducer elements, image reconstruction is required [28]. In comparison to PAM, PACT generally offers high imaging speed and greater penetration capabilities, albeit at the expense of lower spatial resolutions. Over the past few years, both PAM and PACT have been widely employed to visualize vasculature spanning micrometer to centimeter scales and extract pulsatile signals in various imaging applications [29,30,31]. Table 1 summarizes the key performance characteristics of different modalities for pulsatile detection. Due to their differences in spatial resolution, the following context will be split into PAM and PACT sections.

## 2. Photoacoustic Pulsatile Signal Extraction and Applications

### 2.1. PAM-Based Dynamic Photoacoustic Imaging

This section summarizes recent advances in pulsatile signal detection and applications using PAM systems. While PPG systems can extract hemodynamic signals from tissue using both visible and near-infrared (NIR) light sources, the strong optical absorption of visible light limits their penetration depth, and the light scattering reduces spatial resolution. Compared to the PPG system, the PAM system capitalizes on the photoacoustic effect and therefore breaks through the scattering limit to capture hemodynamics in a deeper region. Thanks to the advancement in high-speed scanning, high PRF light source and high-throughput data acquisition, PAM systems are able to sample the heart rate waveform at a sampling frequency far exceeding the required rate. Moreover, PAM enables detailed oxygen saturation (sO_2_) mapping with high spatiotemporal resolution. By utilizing PAM systems, various groups were able to reveal the hemodynamics underneath the human dermal layers, extracting pulsatile signals from a specific vessel region. In high-speed PAM imaging systems, studies further revealed blood oxygenation dynamics in pulsatile arteries. In addition, blood pulse wave velocity measurement was proposed by measuring the electrocardiogram (ECG) and local blood flow velocity using PAM.

#### 2.1.1. Measurement of Heart Rate Waveform and Vascular Dynamics

Ahn et al. [32] used high-resolution and high-speed PAM to monitor vascular dynamics in human fingers. The authors demonstrate the ability of PAM to visualize and measure various parameters of microvasculature networks in human finger. They specifically focus on monitoring the position displacement of blood vessels associated with arterial pulsation and quantifying oxygen saturation and blood perfusion during and after arterial occlusion. It should be noted that the extracted pulsatile signal primarily originates from axial displacement, which is consistent with observations from ultrasound imaging [33]. The proposed system is modified from a commercial PAM and employs two nanosecond pulsed lasers with wavelengths of 532 nm and 559 nm to measure oxygen saturation. The generated PA waves were captured by a 50 MHz ultrasound transducer mounted on motorized linear stages to expand the field of view. The system achieved lateral and axial resolutions of 5 μm and 30 μm, respectively, and an imaging speed of 50 Hz, which is suitable for capturing the heart rate.

**Figure 1:**
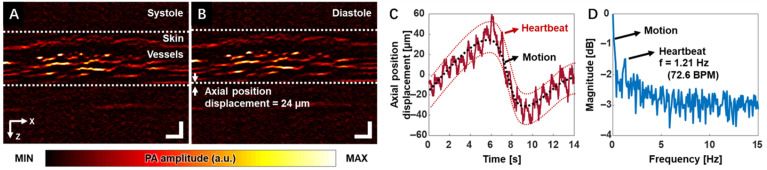
PAM-based monitoring of arterial pulsation and oxygen saturation in a human finger (reproduced with permission from Ref. [32]). Cross-sectional PA image of finger at two statuses, systole (**A**) and diastole (**B**). Axial displacement of the finger vessel (**C**) and its corresponding frequency spectrum (**D**).

Figure 1A,B demonstrate the PA B-mode images of the finger during the systole and diastole phases, respectively. Skin and vessel layers can be clearly visualized. Figure 1C,D show the blood vessels’ position displacement over time and the corresponding frequency spectrum, respectively. The subject’s heart rate can be clearly captured and represented in the frequency spectrum. It should be noticed that the heart-rate waveform was extracted explicitly from a desired region of interest, which demonstrates the potential for precise local hemodynamic monitoring. These findings demonstrate that high-resolution functional PAM can be a valuable tool in assessing vascular dynamics during peripheral vascular examinations, with the potential to measure parameters such as heart rate, oxygen saturation, and blood perfusion.

**Figure 2:**
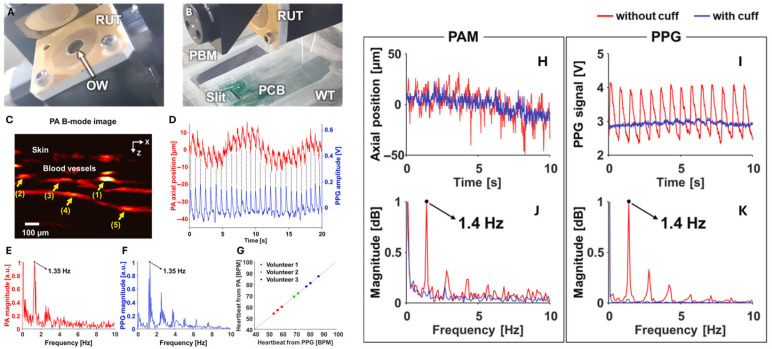
Acquisition of Pulsatile Signals from PAM B-mode Imaging and PPG (reproduced with permission from Ref. [34]). (**A**) A customized ring-shaped transducer. (**B**) PPG PCB aligned with the scanning probe. RUT, ring-shaped US transducer; OW, optical window; PBM, parabolic mirror; and PCB, printed circuit board. (**C**) Cross-sectional PA B-mode image of a human finger, highlighting five blood vessels (yellow arrows) tracked for averaged movement quantification. (**D**) Time-dependent vascular displacement (PAM) and PPG signal variations. (**E**,**F**) Frequency spectra of the PA and PPG waveforms in (**D**), respectively, both showing a dominant peak at 1.35 Hz. (**G**) Heart rate comparison between vascular movement (PAM) and blood volume changes (PPG) across three healthy volunteers (three trials each). (**H**,**I**) Vascular dynamics (PAM) and PPG signals under normal and brachial cuff-induced conditions. (**J**,**K**) Corresponding frequency responses of (**H**,**I**).

In a subsequent study, the same group [34] developed another integrated system combining PAM and PPG to understand hemodynamic features in detail. As a commonly used clinical tool, PPG provides visualization of the hemodynamic waveforms. However, PPG could not provide any images of the blood vessel. The proposed fully integrated PAM-PPG system enabled simultaneous acquisition of vascular images (by PAM) and hemodynamic waveforms (by PPG) from human fingers. The OR-PAM system employed a 532 nm pulsed laser to generate photoacoustic signals, which were detected by a customized 15 MHz ring-shaped ultrasound transducer (shown in Figure 2A). A PPG printed circuit board (PCB) composed of a 532 nm LED and a photodiode is aligned with the PA probe described above (shown in Figure 2B). Authors also programmed the sampling intervals of PAM and PPG to synchronize the data acquisition sequence. The maximum scanning speed of two-dimensional (2D) PA imaging is 100 Hz to meet the sampling requirements of the heartbeat.

Results from the system are shown in Figure 2. Skin and vessel can be clearly observed in Figure 2C, the acquired vessel movements from PPG signals are shown in Figure 2D, which match well with each other. Figure 2E,F show the frequency domain spectrum of vessel movement and PPG signal, respectively. PPG signals acquired from three subjects agree well with vessel movement obtained from PAM (shown in Figure 2G). The presented results indicate that the integrated PAM-PPG system is capable of simultaneously capturing PA images and PPG signals. The vascular images and PPG signals from live human finger were continuously recorded to validate the system’s efficacy. Heart rate (HR) from the vascular movement, as detected by PAM, is derived, and directly compared with the one derived from blood volume changes. The two results exhibited strong consistency. Consistent with prior findings, the pulsatile signals detected by PAM originate exclusively from vessel movement rather than changes in PA intensity within a localized region. Figure 2H demonstrates the vessel position variation and Figure 2I shows PPG signals with and without arterial occlusion marked with red and blue line, respectively. The corresponding frequency domain spectra are shown in Figure 2J,K. The results indicate that the hemodynamic changes mentioned above were not observable during arterial occlusion when blood flow was temporarily blocked. Leveraging the monitoring of hemodynamic variations in deep tissue, the proposed system offers the potential to accurately differentiate between arteries and veins, enabling precise arterial mapping within the imaging region. This capability is made possible by advancements in high spatial and temporal resolution PAM, which analyzes subtle distinctions in blood flow dynamics, and pulsatile characteristics between arterial and venous structures.

#### 2.1.2. Measurement of Dynamics in Blood Oxygenation

**Figure 3:**
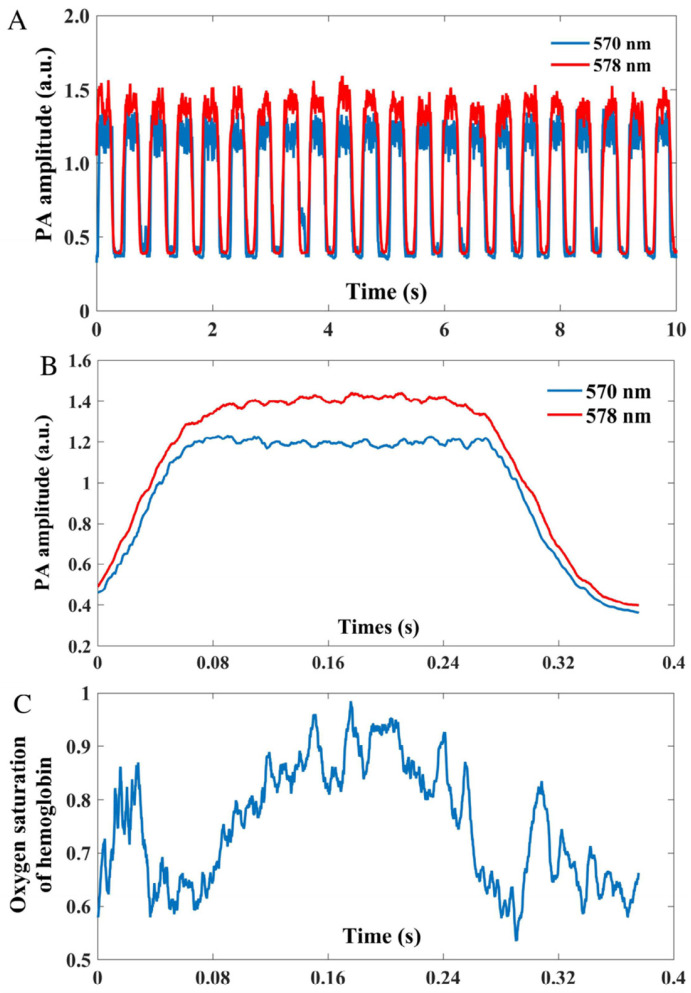
Dynamic PAM amplitude and sO_2_ variations in a selected artery of the cortical vasculature in a cat (re-produced with permission from Ref. [35]). (**A**) PA signal amplitude of the artery at 570 nm and 578 nm acquired over 10 s duration. (**B**) Averaged PA amplitude at 570 nm and 578 nm, with signal alignment performed at amplitude valleys. (**C**) Pulsatile sO_2_ measurements across successive cardiac cycles.

Li et al. presented the use of PAM to study the blood oxygenation dynamics of arteries from animal experiments [35]. The system was constructed based on the confocal OR-PAM design [36]. A 30 MHz ultrasound transducer was coupled to a rhomboid prism to detect PA signals generated by a pulsed dye laser. During imaging, the acoustic lens was submerged in a deionized water tank, the bottom of which was a transparent high-density polyethylene membrane. Unlike previous studies [32,34], pulsatile signal was extracted from both vessel movement and PA amplitude fluctuation. One potential reason is that the artery examined here has a larger diameter, making PA intensity variations caused by blood volume changes more pronounced and easier to detect. The PA signal amplitude waveforms of an artery at two different wavelengths (570 & 578 nm) are depicted in Figure 3A. While dual-wavelength sO_2_ measurements typically use well-separated wavelengths to enhance sensitivity to differentiate absorption of oxy- and deoxy-hemoglobin, this study employed closely spaced wavelengths (570 nm and 578 nm). The 570 nm wavelength lies near an isosbestic point for hemoglobin [37], serving as a normalization or reference channel, whereas 578 nm is closer to the oxy-hemoglobin absorption peak, aiding in the differentiation of the two hemoglobin species [38]. To enhance the accuracy of sO_2_ measurement, the team divided the signal at each wavelength into single-period segments. These segments were then synchronized based on their lowest points to calculate the average signal amplitude over a cardiac cycle, as demonstrated in Figure 3B. The average signal amplitude during each cardiac cycle at the respective wavelengths was utilized to compute the temporal variation in sO_2_. As depicted in Figure 3C, during one cardiac cycle, the sO_2_ in a pulsating artery experiences a rapid increase as fresh blood is pumped through the artery. This increase in sO_2_ corresponds to the rise in blood volume within the vessel before it eventually stabilizes to a steady state. The proposed study highlights the feasibility of measuring dynamic sO_2_ in arteries, which could provide valuable information about oxygen metabolic rate. Besides dynamic sO_2_ monitoring, authors also discuss the potential of PAM in quantifying metabolic rate of oxygen in various organs and its applications in ophthalmic research.

#### 2.1.3. Measurement of Pulse Wave Velocity

Yeh et al. [39] discussed the measurement of blood pulse wave velocity (PWV) using PAM. PWV is an important indicator of vascular stiffness and is commonly used in evaluating vascular diseases. Traditional imaging modalities have limitations in measuring PWV, but PAM offers a noninvasive and high-resolution solution. The proposed system comprises an OR-PAM system and a custom-made ECG recorder. In the system, two lasers were combined using a beam splitter to enable pulse-to-pulse wavelength switching for measuring sO_2_. The generated PA wave was focused by an acoustic lens, detected by an unfocused ultrasonic transducer, and amplified by two cascaded electrical amplifiers. Simultaneously, ECG were recorded using three electrodes—one connected to the ground, one to the front leg, and one to the hind leg of a mouse. The ECG signals were amplified by a high-gain differential amplifier. Both the acquired PA and ECG signals were digitized using a dual-channel high-resolution digitizer and stored in a computer for subsequent offline data processing.

**Figure 4:**
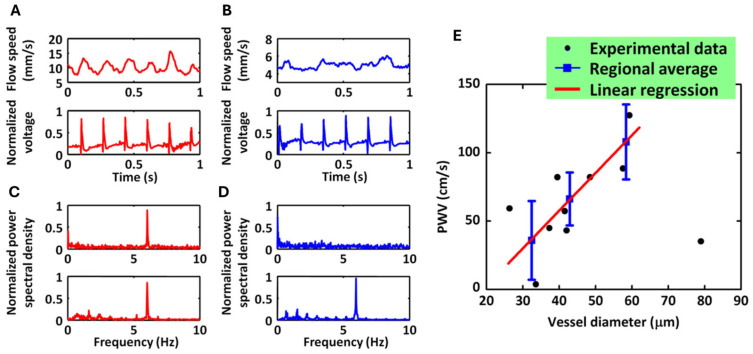
PAM-based quantification of hemodynamic responses in arteries and veins in response to cardiac pulsation (re-produced with permission from Ref. [39]). (**A**) Arterial flow velocity (**top**) and synchronized ECG (**bottom**). (**B**) Venous flow velocity (**top**) and synchronized ECG (**bottom**). (**C**) Normalized power spectra of arterial flow (**top**) and ECG (**bottom**) from (**A**). (**D**) Normalized power spectra of venous flow (**top**) and ECG (**bottom**) from (**B**). (**E**) Pulse wave velocity (PWV) as a function of vessel diameter across ten vascular segments.

The team first identified major arteries and veins within a 5 × 2.5 mm^2^ region of interest in a mouse ear using dual-wavelength (532 & 563 nm) OR-PAM measurement. They then selected two cross-sections, one from an artery and one from a vein, for a 30 s OR-PAM monitoring of blood flow while simultaneously recording an electrocardiogram (ECG). Representative one-second segments of the recorded blood flow and ECG patterns in the artery and vein are displayed in Figure 4A,B, respectively. The Fourier analysis of the entire 30 s blood flow pattern and ECG revealed a notable pulsation-induced oscillation tone in the arterial blood flow, as depicted in Figure 4C. Conversely, such oscillations were absent in the venous flow, as shown in Figure 4D. Therefore, the PWV measurement was only conducted in peripheral arteries and arterioles. Figure 4E demonstrated the measured PWV versus vessel diameters. A linear correlation between the PWV and the vessel diameter was observed. These findings highlight the potential of PAM for precise in vivo measurement of PWV.

### 2.2. PACT-Based Dynamic Photoacoustic Imaging

This section discusses the recent applications of pulsatile signal measurement using PACT. Overall, PACT systems offer a larger field of view than PAM systems. It can image different organs and differentiate arteries and veins based on the pulsatile amplitude distribution in the image. Moreover, by combining ultrasound technique, the artery position can be precisely allocated using information from both modalities.

#### 2.2.1. Measurement of Cardiac Dynamics with Volumetric PACT in an Isolated Heart

**Figure 5:**
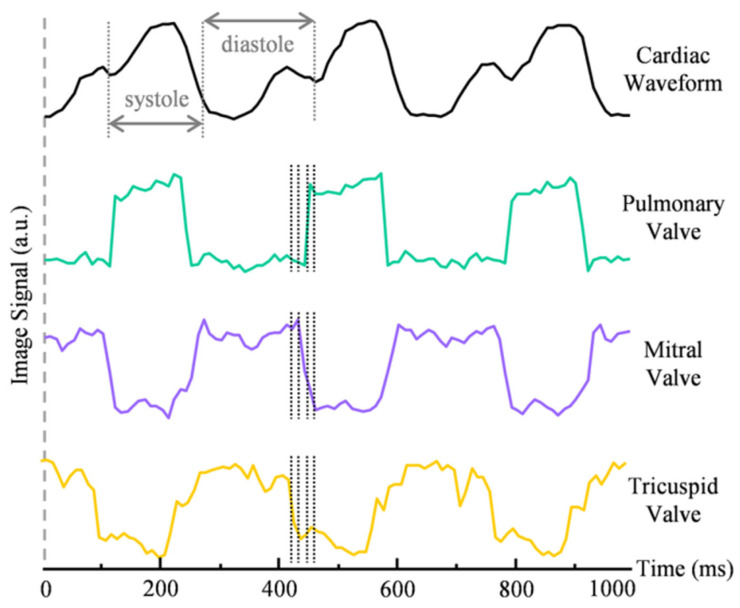
Time-resolved signal profiles from a selected voxel in the PACT image (reproduced with permission from Ref. [40]).

Lin et al. [40] present an ultrafast volumetric photoacoustic imaging system for visualizing a Langendorff-perfused heart. The system excites PA signals using an 800 nm laser at a 100 Hz PRF, which are then captured by a spherical matrix ultrasound array and reconstructed into volumetric images. The system provides a large field of view (FOV) to comprehensively capture cardiac motion while maintaining sufficient spatial resolution to resolve fine cardiac structures. By leveraging the high-frame-rate (100 Hz) volumetric imaging capability, detailed heartbeat dynamics can be extracted through temporal analysis of the reconstructed image sequences. Additionally, the inherent depth advantage of photoacoustic imaging is further enhanced by the Langendorff perfusion protocol, which replaces blood with clear perfusate. This configuration minimizes optical attenuation from blood, allowing the residual hemoglobin in the myocardium to serve as the primary contrast mechanism. As a result, the system achieves detailed visualization of regional cardiac muscle activity throughout the heart. Figure 5 demonstrates these capabilities, showing both the cardiac waveform and signal intensity profiles from voxels selected at the pulmonary, mitral, and tricuspid valves. This performance enables not only real-time monitoring of cardiac mechanics but also reveals functional and pathological features previously challenging to capture in intact hearts.

#### 2.2.2. Real-Time Measurement of Pulsatile Dynamics in Human

Song et al. [33] developed a high-resolution PA imaging system by using a transducer array with 30 MHz central frequency. The proposed system demonstrates the feasibility of using a PACT to noninvasively image human pulsatile dynamics in vivo. The system enables real-time B-scan imaging at 50 Hz and high-speed three-dimensional (3D) imaging to monitor pulsatile motion and sO_2_ changes in a human wrist artery, providing the first real-time PAI of human physiological dynamics. To maximize the PA sensitivity, authors used light at 570 nm to excite PA signal, where oxy- and deoxy-hemoglobin molecules have the same molar optical absorption coefficient. To couple the generated PA signal with an ultrasound transducer array, a water container filled with deionized water and a low-density polyethylene (LDPE) film window were used, minimizing direct contact with the skin surface. This system provides real-time imaging of microvasculature in humans with a large field of view, capturing hemoglobin changes and arterial pulsations. These results open up new avenues for studying physiological dynamics in preclinical and clinical settings.

**Figure 6:**
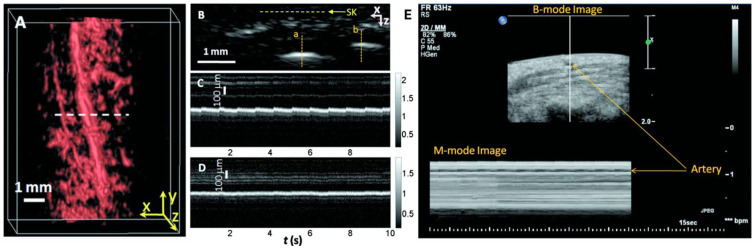
Dynamic in vivo photoacoustic and ultrasound imaging using a PACT system (reproduced with permission from Ref. [33]). (**A**) Volumetric photoacoustic image of a human hand, with dashed line indicating the cross-section for real-time B-scan imaging. (**B**) B-scan image corresponding to the horizontal dashed line in (**A**), showing skin surface (SK). (**C**) M-mode image along vertical line a in (**B**), demonstrating time-resolved arterial pulsation. (**D**) M-mode image along vertical line b in (**B**), showing venous dynamics. (**E**) Corresponding ultrasound images of arterial pulsation.

The team conducted scans on a palm region near the wrist where a prominent artery at 1 mm deep was used to investigate pulsatile dynamics as shown in Figure 6A. The 3D image was first captured and then the scanning probe was affixed to conduct a real-time B-scan lasting 10 s (see Figure 6B). Due to the strong light absorption of hemoglobin at 570 nm, the lower portion of the artery was less visible. Nevertheless, the team successfully recorded the motion dynamics of arterial pulsation. An M-mode image through the artery’s center is displayed in Figure 6C. The estimated pulsatile rate from this image was 66 beats per minute, consistent with the 65 ± 2 beats per minute measured by a pulse oximeter. For comparison, an M-mode image of a vein was shown in Figure 6D, which disclosed relatively weak motion in the vein, presumably induced by skin movement caused by arterial pulsation. The team also noted that in the ultrasound image (Figure 6E), the M-mode image revealed that the artery predominantly expanded in a direction perpendicular to the skin surface, with only minimal expansion observed parallel to the skin surface. Although both PA and US M modes can detect pulsatile activity, the waveform origins differ. Pulsatile signals from PA M mode included both arterial position vibration and optical absorption, while those of US M mode were only induced by tissue movement from the same region.

#### 2.2.3. Quantification of Arteries and Veins in Human Breast Based on Pulsatile Signal

Lin et al. [41] developed a single-breath-hold PACT (SBH-PACT), for high spatiotemporal resolution PA imaging with large field of view. SBH-PACT overcomes the limitations of previous breast PACT systems by achieving sufficient penetration depth, high spatial and temporal resolutions, minimal limited-view artifacts, and high sensitivity to detect breast masses. The system uses 1064 nm light illumination and a 512-element full-ring ultrasonic transducer array to obtain a volumetric 3D image of the entire breast within a single breath-hold (~15 s). The high imaging speed also enabled dynamic studies such as PA elastography to further improve tumor detection. The system employs 1064 nm light and 2.25 MHz unfocused ultrasonic transducer array, enabling up to 4 cm in vivo imaging depth. Second, SBH-PACT incorporates one-to-one element to channel amplification and data acquisition circuits, allowing it to acquire a full 2D cross-sectional breast image with a single laser pulse or create a volumetric 3D image of the entire breast within a single breath-hold of 10 s. Its 10 Hz 2D frame rate permits the observation of biological dynamics in a cross-section.

**Figure 7:**
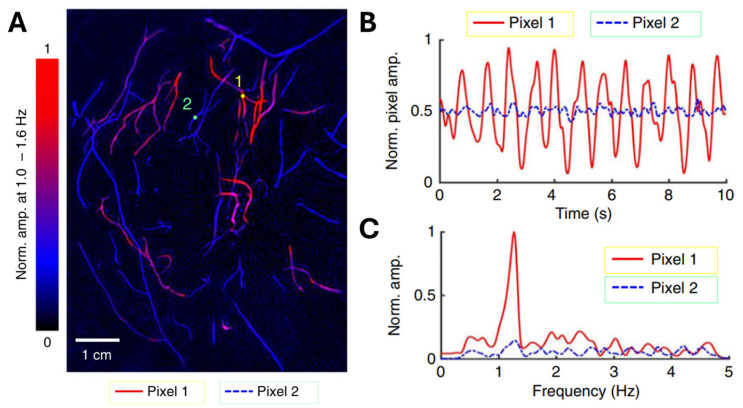
Pulsatile mapping in a PACT-based breast imaging system (reproduced with permission from Ref. [41]). (**A**) Heartbeat-encoded vascular mapping of a breast cross-section (arteries: red; veins: blue). (**B**) Time-domain amplitude variations at two selected pixels (yellow and green markers in panel a). (**C**) Frequency spectrum of pixel-value fluctuations from (**B**), showing arterial pulsation at ~1.2 Hz (heart rate).

Using SBH-PACT in 2D mode with a 10 Hz frame rate, the team continuously monitored the pulsatile deformation of arteries inside the breast by fixing the transducer array at a specific elevation. After that, the PA signals were analyzed pixel by pixel in the frequency domain to distinguish arteries and veins based on the heartbeat frequency. As shown in Figure 7A, the authors chose a single pixel from an artery and another from a vein, denoted by circular markers 1 and 2, respectively. They then quantified the variation in pixel values as shown in Figure 7B. The consistent cyclic patterns observed in the artery pixel values suggest that these changes can be attributed to the propagation of pulse waves within the arterial network. In contrast, those vessels without pixel value variation belong to the venous network. Moreover, the frequency of these oscillations, as depicted in Figure 7C, agrees with the subject’s heart rate at approximately 1.2 Hz. Leveraging the high spatial and temporal resolutions, a depth-resolved pulsatile mapping from the scanning region can be derived to identify arteries and veins, respectively. The uniqueness of this PA imaging technique enables vessel category differentiation at a specific depth.

Moreover, given that arterial blood typically maintains a relatively narrow range of sO_2_, it is possible to leverage average PA signals obtained from arteries as a guide star to calibrate local optical fluence deep within the breast. This calibration process can facilitate the precise quantification of functional parameters using multi-wavelength PACT.

#### 2.2.4. Measurement of Pulsatile Signals Using Dual Modal Ultrasound and Photoacoustic Computed Tomography

Zhang et al. [42] reported a video-rate dual-modal imaging platform that combines wide-beam harmonic ultrasound (WBHUS) and PACT. The system uses a clinical-grade linear-array transducer for both animal and human imaging. The harmonic US imaging provides high-resolution anatomical references to locate PA features and also enhances PA image quality by reducing artifacts. To achieve high imaging speed, the system employs a collaborative scheme of wide-beam transmission and pulse phase inversion. The system enables high-resolution co-registered US/PA tomographic imaging and single-breath-holding 3D imaging, and has been demonstrated for dual-contrast anatomical imaging, visualizing interventional procedures, and monitoring hemodynamics in animals and humans.

The platform uses a clinical-grade linear-array transducer and aims to improve the imaging quality and speed of ultrasound/photoacoustic imaging. A laser with 410–2500 nm tunable range and 20 Hz PRF is applied for PA imaging. The harmonic ultrasonography employs pulse phase inversion to reduce clutter and improve spatial resolution, while wide-beam ultrasound transmission enables a 20 Hz imaging rate with better image quality than plane wave imaging. By combining these two techniques simultaneously, the system effectively achieves high-resolution anatomical imaging while monitoring hemodynamics in both animals and humans through integrated dual-modal ultrasound and PA imaging.

**Figure 8:**
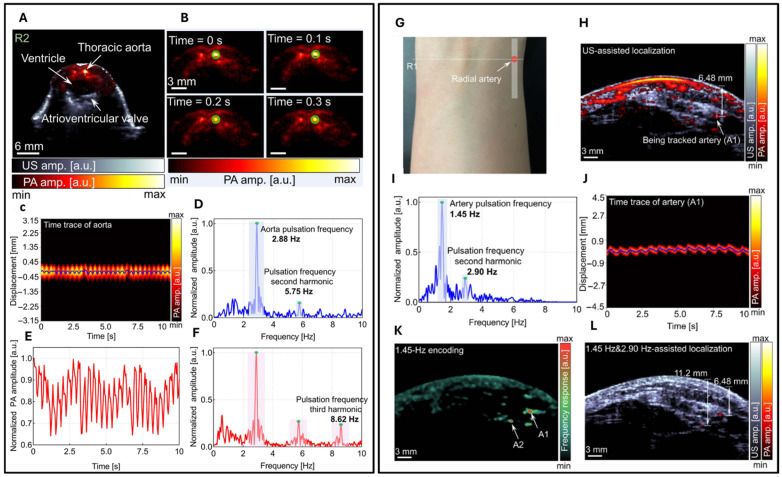
Dual-modal harmonic US and PACT imaging of mouse and human vasculature (re-produced with permission from Ref. [42]). (**A**) Co-registered harmonic US/PA cross-sectional image of liver. (**B**) Time-series PA images of thoracic aorta through one cardiac cycle. (**C**) Aortic displacement along vertical line in (**A**) with peak amplitudes (blue line). (**D**) Frequency spectrum of (**C**) showing fundamental and second-harmonic components. (**E**) PA amplitude dynamics of tracked aorta in (**B**). (**F**) Frequency spectrum of (**E**) revealing fundamental to third-harmonic frequencies. (**G**) Photograph of measurement site. (**H**) Co-registered US/PA image identifying a 6.48 mm deep artery. (**I**) Frequency spectrum of arterial signal showing cardiac harmonics. (**J**) Temporal displacement profile (blue line) along marked vessel in (**H**). (**K**) Heartbeat-encoded (1.45 Hz) pseudo-color PA map. (**L**) Composite US and segmented PA arterial image.

The authors initially validated the system by imaging mice. In Figure 8A, co-registered US and PA images are presented for a cross-sectional slice near the heart. Representative PA images throughout one heartbeat cycle are illustrated in Figure 8B. The variations in signal position, as captured in Figure 8C, are represented by the vertical line in Figure 8A, accompanied by a Fourier analysis illustrated in Figure 8D. Furthermore, the author identified a distinct, rhythmic pulsation in the amplitude of the aorta, as demonstrated in Figure 8E, with its corresponding Fourier analysis presented in Figure 8F. These variations are synchronized with the heartbeat and thus serve as indicators of cardiovascular dynamics. The frequency of both displacement and amplitude associated with the heartbeat was measured at 2.88 Hz, demonstrating consistent alignment between the two parameters. The system’s high sensitivity also allowed the detection of the second (5.75 Hz) and third harmonics (8.62 Hz) of the heartbeat. The result indicates that with high imaging speed, a precise pulsatile waveform can be captured, therefore revealing more hemodynamic features. Subsequently, the authors applied the same system to image the human forearm. A continuous monitoring of a cross-sectional forearm area is illustrated in Figure 8G. Using dual-modal imaging, the radial arteries were successfully located, as displayed in Figure 8H. The authors recorded changes in arterial position, represented by the white solid line in Figure 8I, and determined the fundamental and harmonic frequencies of the major radial artery through Fourier transformation. Furthermore, an analysis of the temporal frequency of each pixel in the PA images allowed for the encoding of blood vessels with pseudo-color, as demonstrated in Figure 8K. Two specific locations exhibiting strong pulsations were identified and marked in Figure 8K and highlighted in the combined ultrasound and PA image overlay, as shown in Figure 8L. The quantitative in vivo results demonstrate significant potential for advancing cardiovascular-related applications. These findings suggest that the technology could enhance the diagnosis, monitoring, and treatment of cardiovascular conditions by providing more accurate and detailed insights into blood flow dynamics, and vascular health.

## 3. Discussion and Conclusions

The detection of pulsatile signals plays a critical role in both clinical and non-clinical applications, particularly for monitoring physiological parameters like heart rate and blood oxygen saturation. This review highlights key advancements in the field, comparing traditional techniques such as PPG and ultrasound with more recent approaches like PA imaging. The findings point to the promise of PA imaging in overcoming limitations inherent to optical and ultrasound methods by offering deeper tissue penetration, higher spatial resolution, and more accurate hemodynamic data acquisition. PPG’s reliance on optical absorption differences makes it ideal for monitoring blood volume changes. The visible wavelength, commonly employed in PPG, limits penetration to superficial tissues owing to strong optical absorption by hemoglobin and melanin [43,44]. While NIR PPG achieves greater penetration depth due to reduced optical absorption, its limited spatial resolution hinders the discrimination of deeper pulsatile signals from superficial tissue contributions [34]. On the other hand, ultrasound-based techniques enable deeper tissue penetration but face challenges in contrast resolution. Because red blood cells are weak scatterers of ultrasound, flow-related pulsatile signals have limited sensitivity, and small-vessel hemodynamics are difficult to assess reliably. As a result, ultrasound often infers pulsatility from vascular wall or tissue motion rather than directly from microvascular blood flow. Furthermore, unlike optical methods, ultrasound lacks sensitivity to hemoglobin absorption, making it unsuitable for measuring local hemoglobin concentration or oxygenation.

In contrast, PA imaging, which combines optical contrast with ultrasound spatial resolution, presents a promising solution. Unlike PPG, PA imaging can detect pulsatile signals from deep tissue, providing both high spatial and temporal resolution. Also, unlike ultrasound, PA imaging can differentiate oxy-hemoglobin and deoxy-hemoglobin and hence quantify pulsation from arteries and veins and measure sO2 at various depths. As an emerging imaging modality, photoacoustic imaging demonstrates great potential for applications in preclinical and clinical studies. By combining plaque lipid spectral signatures [45,46] with temporal information derived from pulsatile signals, PAI enables a more comprehensive assessment of vascular status. Pulsation detection, in particular, facilitates accurate sO_2_ quantification in deep tissue by reliably differentiating arteries from surrounding vasculature. Because arterial blood exhibits a relatively narrow range of sO_2_, photoacoustic signals from arteries can be used to calibrate local fluence, thereby improving the precision of sO_2_ measurements [41]. This capability benefits translational clinical application, including but not limited to breast cancer detection [47,48], functional brain imaging [49,50] and hemodynamic analysis in cardiovascular studies [51]. Beyond these examples, pulsation also serves as a unique biomarker for liveness detection in PA biometric imaging, since spoof or prosthetic hands lack pulsative signals [52]. In addition, PAI has been successfully applied to evaluate tissue perfusion in chronic leg ulcers [53,54], while pulsatile mapping could offer additional information about tissue perfusion [42]. By integrating chemical, structural, and functional information, PAI can simultaneously provide a comprehensive assessment of tissue status, offering significant potential for the diagnosis and management of perfusion-related conditions.

Despite the many advantages of photoacoustic imaging, there are still challenges hindering its clinical translation. One major limitation is the trade-off between penetration depth and spatial resolution, which restricts high-resolution imaging to relatively shallow tissues and reduces detail when probing deeper structures [55]. The technical complexity and high cost of clinical systems, which often require powerful pulsed lasers, sensitive ultrasound detectors, and advanced acquisition hardware, also limit scalability and routine deployment. Moreover, artifacts from incomplete data sampling and reconstruction algorithms can degrade image quality and complicate interpretation [31,56]. Biological factors such as optical scattering and acoustic attenuation, particularly in complex structures like the skull, further constrain applications in areas such as brain imaging [55]. Safety standards on laser fluence impose limits on the amount of optical energy that can be delivered, which in turn restricts achievable signal strength and imaging depth [57,58]. Efforts to use compact and lower-cost light sources, such as laser diodes or LEDs, are promising but currently suffer from reduced signal-to-noise ratio unless supported by sophisticated denoising strategies [59,60]. Beyond the technical aspects, there are also important regulatory and standardization barriers [61]. At present, there is no widely adopted framework for consistent image analysis or interpretation, and most clinical studies remain pilot-scale with limited patient cohorts, making it difficult to demonstrate statistical robustness and clear clinical benefit over established imaging modalities [31,62,63]. Together, these limitations illustrate why the clinical adoption of photoacoustic imaging, while promising, still requires further technological optimization, large-scale validation studies, and harmonization of regulatory pathways before it can become a routine diagnostic tool.

Several pressing research directions could accelerate the clinical adoption of pulsatile detection using PA imaging. First, advancements in compact light sources such as high PRF pulsed laser diodes (LDs) and high-power LEDs, coupled with portable ultrasound transducers, can reduce system size and cost, making point-of-care PA-based monitoring feasible [64,65]. Second, AI-enhanced image reconstruction, spectral unmixing, and decision support tools can automate pulsatile signal extraction, improve image quality, robustness against motion artifacts, and assist clinicians in real-time diagnosis [66,67]. Lastly, large-scale, multi-center clinical trials are needed to evaluate the feasibility of pulsatile detection using PAI, establish diagnostic benchmarks. This will ensure consistent interpretation across different hardware and software platforms [31].

In conclusion, pulsatile detection through PAI provides a powerful means of overcoming the limitations of traditional optical and ultrasonic techniques. By combining optical contrast with ultrasonic resolution, PAI enables deep-tissue monitoring of dynamic vascular signals with high spatial and temporal fidelity. This capability extends beyond conventional heart rate and oxygen saturation measurement, allowing precise assessment of blood flow velocity, pulse wave velocity, and arterial–venous differentiation. Pulsatile detection also enhances functional applications, including improved sO_2_ quantification via fluence calibration, perfusion monitoring in peripheral artery disease, and liveness verification in biometric imaging. Looking ahead, advancements in compact light sources, AI-assisted analysis, and multi-center clinical trials will be essential for establishing pulsatile PAI as a standardized, non-invasive tool for comprehensive vascular assessment.

## Figures and Tables

**Table 1:** Comparison of imaging modalities for pulsation detection.

Modality	Imaging Depth	Sensitivity	System Cost	System Size	Field of View	Temporal Resolution
Photoplethysmography	Low	Medium	Low	Low	Low–High	High
Ultrasound imaging	High	Low	Medium	Medium–High	High	Medium
Photoacoustic microscopy	Medium	High	High	Medium	Medium	Medium–High
Photoacoustic computed tomography	High	High	High	High	High	Low–Medium

## Data Availability

Not applicable.

## Abbreviations

2D	Two-dimensional
3D	Three-dimensional
AR-PAM	Acoustic-resolution photoacoustic microscopy
CBP	Central blood pressure
DSPF	Diffuse speckle pulsatile flowmetry
ECG	Electrocardiogram
FOV	Field of view
HR	Heart rate
iPPG	Imaging photoplethysmography
LDPE	Low-density polyethylene
LEDs	Light emitting diodes
NIR	Near-infrared
OR-PAM	Optical-resolution photoacoustic microscopy
PA	Photoacoustic
PACT	Photoacoustic computed tomography
PAD	Peripheral artery disease
PAI	Photoacoustic imaging
PAM	Photoacoustic microscopy
PCB	Printed circuit board
PPG	Photoplethysmography
PRF	Pulse repetition frequency
PWV	Pulse wave velocity
SBH-PACT	Single-breast-hold photoacoustic tomography
sO2	Oxygen saturation
US	Ultrasound
WBHUS	Wide-beam harmonic ultrasound
